# Temperature-Dependent Biofilm Development in Antarctic Endophytic Microbial Communities

**DOI:** 10.3390/microorganisms14030580

**Published:** 2026-03-04

**Authors:** Olga Iungin, Geert Potters, Oleksandr Kalinichenko, Yevheniia Prekrasna-Kviatkovska, Olena Moshynets, Oleksandr Kazakov-Kravchenko, Marina Sidorenko, Olena Okhmat, Saulius Mickevičius

**Affiliations:** 1 Department of Biotechnology, Leather and Fur, Faculty of Chemical and Biopharmaceutical Technologies, Kyiv National University of Technologies and Design, 01011 Kyiv, Ukraine; kalinichenko742135@gmail.com (O.K.); oxmat.oa@knutd.edu.ua (O.O.); 2 Biofilm Study Group, Institute of Molecular Biology and Genetics, National Academy of Sciences of Ukraine, 03143 Kyiv, Ukraine; moshynets@gmail.com; 3 Faculty of Natural Sciences, Vytautas Magnus University, 53361 Kaunas, Lithuania; marina.sidorenko@vdu.lt (M.S.); saulius.mickevicius@vdu.lt (S.M.); 4 Antwerp Maritime Academy CORrosion Team, Nautical Faculty, Antwerp Maritime Academy, 2030 Antwerp, Belgium; 5 Department of Bioscience Engineering, University of Antwerp, 2000 Antwerp, Belgium; 6 Biology and Ecology Department, State Institution National Antarctic Scientific Center, 01601 Kyiv, Ukraine; preckrasna@gmail.com; 7 Biomedical Chemistry Department, Institute of Molecular Biology and Genetics, National Academy of Sciences of Ukraine, 03143 Kyiv, Ukraine

**Keywords:** Antarctic endophytes, microbial communities, biofilm architecture, polar ecosystems, eDNA

## Abstract

Climate change is reshaping Antarctic ecosystems, where the resilience of *Deschampsia antarctica* and *Colobanthus quitensis* is mediated by endophytic microbial communities assembled under strong abiotic drivers. This study explores the temperature-dependent biofilm development in two Antarctic endophytic microbial communities (ALS and LS). Multivariate analysis revealed a fundamental trade-off between planktonic expansion and biofilm matrix investment as a function of thermal cues. While moderate warming (15–25 °C) optimized cell viability and turbidity, extreme thermal stress at 37–42 °C in nutrient-rich conditions triggered a significant shift toward a matrix-rich signature, characterized by a synergistic increase in total DNA and cellulose. Crucially, at the thermal extreme of 42 °C, we observed a decoupling of optical density from culturable biomass, where high turbidity did not translate into viable cells, signaling a state of severe environmental stress. These results identify 25 °C as the quantitative threshold for optimal growth, while temperatures of 37–42 °C act as a specific trigger for protective matrix production. Such thermal plasticity suggests that Antarctic endophytes are evolutionarily primed for persistence not only in cold native niches but also during bird-mediated dispersal at endothermic host temperatures.

## 1. Introduction

Climate change directly impacts the Antarctic Peninsula, facilitating the successful colonization of ice-free areas by two Antarctic vascular plants, *Deschampsia antarctica* and *Colobanthus quitensis* [1]. The resilience of these species is partly attributed to endophytic microorganisms, which function as plant growth-promoting bacteria and form a diverse variety of biofilm structures [2]. These interactions are shaped by extremely low temperatures, limited water availability, and nutrient-poor soils, which foster distinct, host-specific microbial assemblages, which in turn enhance plant survival in Antarctica [3]. Abiotic environmental factors determine the available species pool in a specific region. Recent culture-independent surveys indicate that bacterial diversity in the rhizosphere of *Deschampsia antarctica* and *Colobanthus quitensis* is more strongly governed by soil physicochemical properties than by plant identity, highlighting abiotic filters as primary drivers of microbial community assembly in Antarctic soils [4]. Consequently, this dominance of abiotic filtering justifies our focus on temperature as the primary environmental driver, allowing us to examine whether extreme thermal shifts override host-specific traits in shaping microbial development. Moreover, the harsh abiotic filters favor psychrophilic and endemic taxa such as *Antarctomyces* spp. and Antarctic *Clostridium* isolates, which are thought to provide functional traits that enhance plant cold tolerance [3]. However, the transition from environmental filtering to enhanced plant fitness is mediated by the formation of specialized biofilms within different micro-habitats of the host [5]. Endophytic bacteria often encounter diverse physical interfaces, including the air–liquid–surface (ALS) interface, which simulates aerated microenvironments such as stomatal cavities or apoplastic spaces, and the liquid–surface (LS) interface, which mimics submerged conditions typical of vascular tissues [6]. Under varying environmental conditions, gene expression drives the synthesis of different biofilm components—such as the exopolysaccharide matrix, amyloids, and extracellular DNA (eDNA). These components are crucial for ecological fitness. For instance, cellulose provides desiccation resistance, while eDNA facilitates horizontal gene transfer, which potentially expands the functional repertoire of the microbiome [7].

Furthermore, in the maritime Antarctic, plants often grow on ornithogenic soils enriched by seabird guano. The dual nature of these endophytes—exhibiting growth optima at both Antarctic temperatures and endothermic ranges (37–42 °C)—suggests a potential life cycle involving birds as intermediate hosts or dispersal vectors [8,9]. Considering the critical role of microorganisms in plant hardiness and adaptation to extreme conditions, investigating the plant–microbe interactions in this unique region is a highly relevant research objective. This study, therefore, focused on characterizing the structural components within the architectural framework of microbial community biofilms associated with Antarctic vascular plants and the driving forces of their formation in a wide temperature range.

## 2. Materials and Methods

### 2.1. Model Microbial Communities

Bacterial isolates used in this research were obtained from the inner tissues of *D. antarctica* and *C. quitensis* plants collected along the western Antarctic Peninsula during the 24th Ukrainian Antarctic Expedition (January–April 2020) [10].

Overnight cultures were prepared in closed microcosms in liquid Nutrient Broth (NB, HiMedia, Ltd., Maharashtra, India) with shaking at 25 °C and 160 rpm. To determine the density of the microbial suspension, an R092 McFarland Standard Set (HiMedia, Ltd., Maharashtra, India) was used, allowing the density to be adjusted to McFarland Standard 1, corresponding to 3 × 10^8^ colony-forming units (CFU) per cubic centimeter. The suspension density was measured spectrophotometrically to achieve an optical density in the range of 0.4–0.5 OD at a wavelength of 620 nm.

Model microbial communities (MMCs) were established from cultivated chemoorganoheterotrophic bacteria associated with Antarctic vascular plants, according to the biofilm type formed by each isolate (Table 1). Isolates that exhibited ALS interface biofilm phenotype were combined into one microbial community. Correspondingly, isolates producing LS interface biofilms were grouped into another. These phenotypically distinct cellular aggregations correspond to the ecological niches of endophytic bacteria within the plant system. To standardize the starting conditions and ensure equal initial competitive opportunities for each strain within the model community, overnight cultures were mixed in equal proportions based on their adjusted CFU/cm^3^ counts and incubated in static, closed microcosms (vials) with Nutrient Broth (HiMedia, Ltd.) and minimal salt medium (MSM) at temperatures of 4, 15, 25, 37, and 42 °C for 3 and 6 days.

The selection of this wide thermal range reflects the environmental heterogeneity of the maritime Antarctic. While a range of 4–15 °C represents typical seasonal soil temperatures, the higher temperatures (37 °C and 42 °C) were chosen to simulate conditions within the endothermic hosts. The parameters investigated were biofilm strength, biomass growth (productivity), and surface attachment, which constituted the combined biofilm assay [11].

### 2.2. Biofilm Analysis

Biofilm visualization was performed using a Leica TCS SPE Confocal Laser Scanning Microscope (CLSM) system equipped with a coded DMi8 inverted microscope (Leica, Germany) and Leica Application Suite X (LAS X) Version 3.4.1 software. Biofilm architecture was quantitatively assessed through CLSM image analysis, focusing on cellulose and eDNA, which serve as the primary structural scaffold and adhesive matrix, respectively. Due to technical constraints and the growth rate of the isolates, LS biofilms were exclusively analyzed at the 6-day mark. To ensure reproducibility and enable quantitative comparison between samples, as well as to avoid non-specific fluorescence, all images were acquired using fixed parameters; laser intensity (set at 15.0%), detector gain (680–700), and offset (0.0%) were kept constant for each fluorophore across all experimental groups. Background noise was minimized using standardized thresholding settings in LAS X software. The following fluorophores were used for staining:

• Nucleic Acids: Propidium iodide (ex/em 537/618 nm), ethidium bromide (ex/em 532/570 nm), and SYBR Green (ex/em 497/520 nm), and the highly specific heavy eDNA stain 7K (ex/em 488/495–510 nm) [12].
• Polymeric Extracellular Matrix (Cellulose): Calcofluor White (ex/em 380/475 nm).

The samples were not fixed, but a coverslip was placed over the stained samples before imaging. Pixel counts were used as a proxy for biomass and matrix concentration, as the fluorescent signal intensity in standardized CLSM volumes has been shown to correlate significantly with the physical density of the biofilm components [13].

The concentration of eDNA in 3- and 6-day-old biofilms was also determined spectrophotometrically. To minimize the co-extraction of intracellular DNA from lysed cells, biofilm samples (550 µL) were centrifuged at a controlled speed (12,000× *g*), and the supernatant was used for eDNA precipitation. The supernatant (500 µL) was precipitated overnight at −20 °C with 50 µL of 3M sodium acetate (pH 5.2) and 1 mL of 96% (*v*/*v*) ethanol. The precipitated DNA was pelleted by centrifugation (13,000× *g* for 15 min), washed with 1 mL of 70% (*v*/*v*) ethanol, and air-dried at room temperature. Samples were then resuspended in 50 µL of TE buffer, and eDNA concentration was measured using a NanoDrop 2000 spectrophotometer (ThermoFisher Scientific, Waltham, MA, USA). While complete avoidance of cell lysis is challenging in biofilm studies, this protocol prioritized the recovery of the extracellular fraction. No DNase digestion was applied to the supernatant prior to precipitation to preserve the integrity of the naturally released eDNA pool.

### 2.3. Statistical Analysis

Statistical analysis was performed using R (version 4.4.1) embedded in RStudio (version 2025.09.0+387 “Cucumberleaf Sunflower” for Windows), with the help of packages ggplot2, gsheet, GGally, lme4, lmerTest, dplyr, tidyr, vegan, emmeans, and factoextra [14,15,16,17,18,19,20,21,22,23,24]. Linear mixed models (LMMs) were created with package lme4, for each biofilm parameter as a function of the medium, the temperature of cultivation, and the duration of cultivation, with and without considering possible interaction effects. In each case, a basic model without interactions was compared to a full interaction model. The models were compared based on the difference in REML as encoded by the lmer function in R package lme4 [16]. In cases where the model fit was identified as “poor” based on residual diagnostics, the results were interpreted with caution, and these instances are explicitly noted in Section 3 as potential limitations due to high biological variability within the microbial communities.

Pairwise comparisons were performed using the Kenward–Roger method to estimate the degrees of freedom and the Tukey method for *p*-value adjustment. In the pair plots, Pearson’s correlation coefficient was used to express correlation.

## 3. Results

### 3.1. Biofilm Growth Parameters and Structural Components in ALS Microbial Communities

The development of ALS microbial communities was significantly modulated by temperature and nutrient availability, showing non-linear responses across the tested thermal range. Even though the different individual strains in a community each have their own optimal temperature ranges, their collective response within the biofilm is frequently non-linear and governed by complex inter-species interactions, which are further modulated by nutrient availability. Therefore, we present a detailed comparative analysis of the community’s total growth parameters across the five tested temperatures and two distinct culture media. For each of the parameters that were measured, two LMMs were constructed in which medium, temperature, and development time (3 vs. 6 days) were used as explanatory variables. One model did not include interaction terms, while the other did.

**Figure 1:**
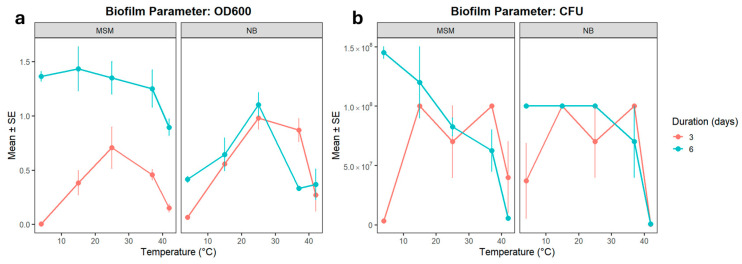
(**a**) Growth rate (OD600) and (**b**) viability (CFU) for ALS-type MMC biofilms in function of temperature, medium, and duration of biofilm development. Bars indicate SE. *n* = 3 for all treatments.

Planktonic growth peaked between 15 °C and 37 °C relative to the 4 °C baseline (Figure 1a). However, the growth dynamics were strongly dependent on the medium and incubation period. Statistical modeling confirmed that the interaction model provided a substantially better fit (REML = 4.8) than the non-interaction model (REML = 53.1), highlighting that the effect of temperature on growth cannot be interpreted independently of the nutrient environment.

Cell viability, measured via CFU counts, followed a similar thermal trend but exhibited much higher variability (Figure 1b). According to the model without interactions, medium composition had no overall effect, nor did a longer incubation time. Variability in CFU counts was substantial, suggesting that the variable is less stable than OD600. When interactions were included, the significant effect of temperature was confirmed, and longer incubation (6 days) also became strongly significant (*p* = 1.91 × 10^−7^), with a negative secondary interaction between the longer duration and NB medium. It should be noted that both linear mixed models for CFU exhibited high residual variation (REML > 1200), indicating that cell viability is less predictable and more sensitive to stochastic biological variation than total optical density. Therefore, these trends should be interpreted with caution regarding precise quantitative predictions.

Comparing OD600 and CFU revealed that growth in the surrounding medium and colony-forming ability respond differently to temperature and environmental conditions. OD600 increased consistently at 15–25 °C and was further enhanced by prolonged incubation, but reduced in Nutrient Broth. CFU counts, by contrast, were highest at the cold baseline and at 15 °C, with a sharp decline at 42 °C. Medium composition did not influence CFU, and incubation effects were weak.

**Figure 2:**
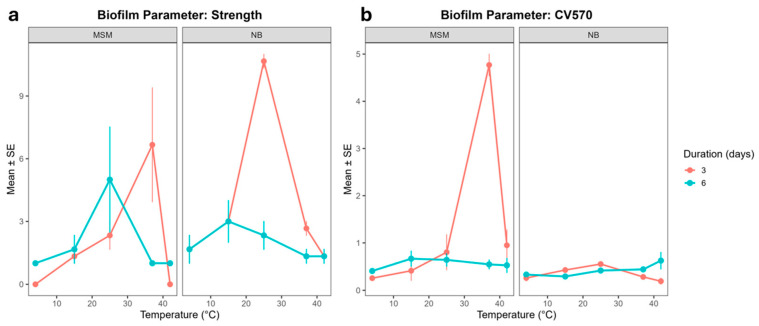
(**a**) Strength and (**b**) attachment (measured as CV570) for ALS-type MMC biofilms in function of temperature, growth medium, and duration of biofilm development. Bars indicate SE. n = 3 for all treatments.

Biofilm strength and surface attachment showed a coordinated response to temperature, both peaking under warmer conditions (25–37 °C), yet their stability was highly sensitive to the nutrient environment and incubation time (Figure 2). The physical robustness of the ALS biofilms was primarily determined by temperature, with maximum strength observed at 37 °C (Figure 2a). At this temperature, the community likely reaches its metabolic peak for matrix production, although this robustness was transient in nutrient-rich conditions. In NB medium, biofilm strength at 37 °C was significantly reduced after 6 days (*p* = 0.004), possibly due to accelerated nutrient depletion or the accumulation of metabolic by-products that trigger biofilm dispersal. Interestingly, at a more moderate 25 °C, NB medium initially boosted biofilm strength, but this effect was lost over time (three-way interaction, *p* < 0.001). This suggests that while rich nutrients accelerate initial biofilm maturation at sub-optimal temperatures, they may lead to premature aging and structural weakening of the biofilm under prolonged incubation.

Surface attachment (CV570) followed a similar thermal trend (Figure 2b), significantly increasing at 37 °C (*p* < 0.001) in minimal media. This indicates that warmer temperatures promote the initial stages of colonization and adhesion. However, we observed a complex context-dependent response involving nutrients and time. At 37 °C and 42 °C, the initial suppression of attachment by nutrient broth was reversed during prolonged incubation (three-way interaction, *p* < 1 × 10^−12^). These quantitative trends in strength and attachment align with the CLSM observations, where the dense, multi-layered architectures seen at 37 °C correlate with the peak values recorded in the mechanical assays.

In contrast, attachment was significantly increased at 25 °C, 37 °C, and 42 °C, but nutrient broth and longer incubation each suppressed attachment at 37 °C. Notably, their combination restored attachment at both 37 °C and 42 °C, revealing a conditional recovery effect absent in the strength trait. Thus, while both traits are stable at the cold baseline and enhanced under warmer, non-native conditions, biofilm strength is primarily shaped by medium-dependent modulation, whereas attachment is more sensitive to the combined effects of medium and incubation duration.

**Figure 3:**
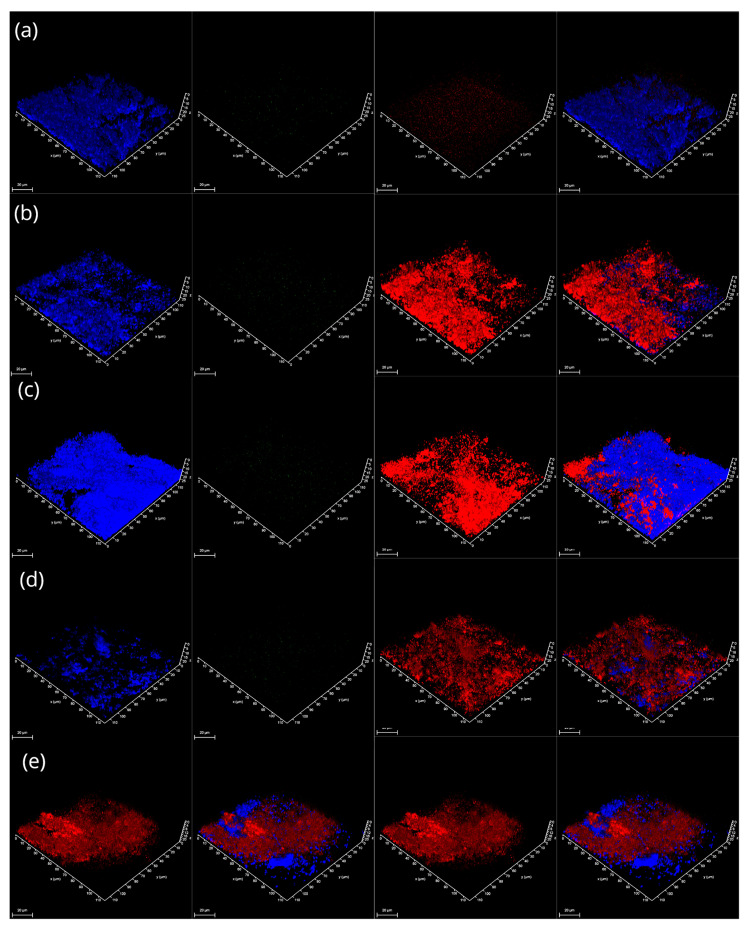
Visualized structure of ALS-type MMC biofilms, after 6 days of cultivation in NB medium at a temperature of (**a**) 4 °C; (**b**) 15 °C; (**c**) 25 °C; (**d**) 37 °C; (**e**) 42 °C. The red signal corresponds to total DNA, green to eDNA, and blue to cellulose, visualized with Calcofluor White. The plot at the far-right side represents the merged signal. Scale bar is 20 μm.

**Figure 4:**
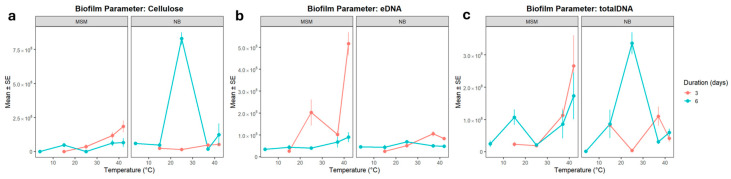
(**a**) Planktonic growth rate (OD600), (**b**) planktonic cell viability (CFU) and (**c**) attachment (expressed as crystal violet retention, measured at 570 nm) of the ALS biofilms in function of temperature, growth medium, and duration of biofilm development, based on quantification of the pixels in the CLSM images (Figure 3). Bars indicate SE. n = 3 for all treatments.

Strength and attachment levels could be explained by the secretion of higher amounts of cellulose and eDNA as structural components of biofilms. Quantitative analysis of CLSM images (Figure 3 and Figure 4) revealed that temperature shifts drive a fundamental reorganization of the biofilm matrix, with cellulose and extracellular DNA (eDNA) showing divergent regulatory patterns. Cellulose production was strongly modulated by temperature, showing a distinct thermal switch between 25 °C and 37 °C. At 25 °C, the combination of nutrient-rich medium and prolonged incubation triggered a striking synergistic increase in cellulose (*p* ≈ 0.10 and *p* ≈ 0.076, respectively). This suggests that at temperatures typical of the Antarctic summer or the external surfaces of the host, the community invests in a robust cellulose-rich framework to enhance structural stability and desiccation resistance.

By contrast, at 37 °C, the same combination of factors significantly reduced cellulose production (*p* = 0.028). This reduction is biologically consistent with the temperature-dependent repression of cellulose biosynthesis operons (e.g., *bcs*) often observed in bacteria during the transition to an endothermic host environment. In this context, the downregulation of cellulose at 37 °C and 42 °C indicates a shift in biofilm strategy. While in the animal/bird-body environment, the community may prioritize rapid proliferation or the synthesis of other attachment factors like amyloids over the energy-intensive production of a rigid cellulose matrix.

eDNA and total DNA displayed distinct responses to environmental cues, suggesting that matrix-associated DNA release is regulated independently of total community biomass. The eDNA pool was notably sensitive to combined stressors. While eDNA was present at the cold baseline (4 °C), its levels significantly increased under extreme conditions (42 °C in MSM, *p* = 0.039). This spike at 42 °C likely reflects stress-induced cell lysis or active secretion, with eDNA acting as a critical structural stabilizer when other matrix components are suppressed. Conversely, nutrient-rich conditions and prolonged incubation consistently reduced eDNA levels. The interaction models (REML = 876.9) confirmed that eDNA release is vulnerable to the synergistic destabilizing effects of high nutrient availability and elevated temperature, which may favor a more transient biofilm structure with reduced matrix-associated DNA.

In contrast to the eDNA fraction, total DNA levels remained more stable or increased under warming. Significant increases were observed at 42 °C (*p* = 0.0009) regardless of the medium or duration, reflecting an overall increase in cellular biomass. A striking three-way synergy was observed at 25 °C (*p* = 0.014), where the combination of NB medium and 6-day incubation produced a peak in total DNA.

The divergence between these two traits highlights that eDNA release is not a simple function of total biomass. While total DNA responds positively to synergistic environmental conditions at 25 °C, the eDNA fraction is more sensitive to suppression. This suggests that the extracellular and intracellular DNA pools are governed by distinct physiological triggers; for instance, total DNA reflects community growth and compensatory stabilization, while eDNA release is a specific architectural response to environmental stress and nutrient availability.

Further overall comparison of the different parameters in a pairwise plot (Figure S1) shows that cellulose content is strongly and positively correlated with total DNA (R = 0.753), but not with strength (R = −0.051) or biofilm attachment (CV570, R = 0.079), and, surprisingly, also not with eDNA (R = 0.190). Strength and CV570 are weakly correlated (R = 0.306), as well as CFU and OD600 (R = 0.366).

### 3.2. PCA Analysis for Biofilm Characteristics

**Figure 5:**
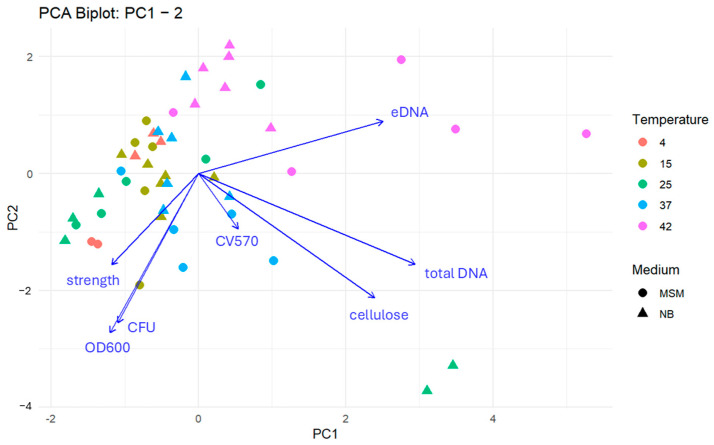
Integrated PCA-biplot for the first and second principal component illustrating the relationship of the different characteristics of ALS microbial communities. Each point represents a sample characterized by cultivation temperature (colour) and medium type (shape). Arrows indicate the loadings of individual biofilm traits, with direction and length reflecting their contribution to the principal components. Traits oriented in similar directions are positively correlated, while opposing vectors suggest negative associations. Samples located near a given vector are associated with higher values of that trait.

**Figure 6:**
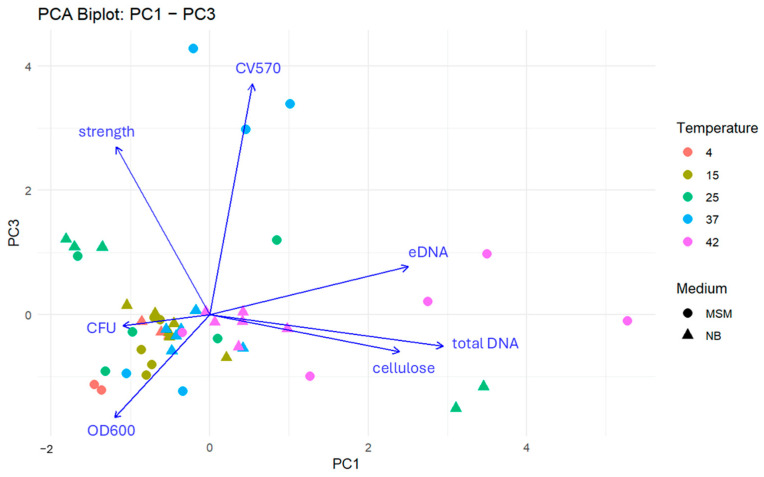
Integrated PCA-biplot for the first and third principal component illustrating the relationship of the different characteristics of ALS microbial communities. Each point represents a sample characterized by cultivation temperature (colour) and medium type (shape). Arrows indicate the loadings of individual biofilm traits, with direction and length reflecting their contribution to the principal components. Traits oriented in similar directions are positively correlated, while opposing vectors suggest negative associations. Samples located near a given vector are associated with higher values of that trait.

To integrate the complex interactions between biotic and abiotic factors, a Principal Component Analysis (PCA) was performed (Figure 5 and Figure 6). The first three components explained 71.0% of the total variance, revealing three coherent, biologically meaningful blocks in the community response. The matrix block (PC1—30.6%) is defined by total DNA, eDNA, and cellulose. High positive scores on PC1 identify “matrix-heavy” biofilms. This signature was most pronounced under both optimal growth at 25 °C (NB, 6 days) and extreme thermal stress at 42 °C (MSM, 3 days). The planktonic and matrix development block (PC2) contrasts planktonic abundance (OD600, CFU) against matrix markers. Samples at 25 °C (NB, 6 days) showed a strong negative PC2 orientation, reflecting a unique state where high planktonic density coexists with intensive matrix production. Conversely, most samples at 42 °C shifted toward positive PC2 scores, suggesting a state in which matrix components (likely from cell lysis) dominate over viable planktonic populations. The structural integrity block (PC3—18.5%) captures mechanical strength and surface attachment (CV570). Its orthogonality to the DNA/matrix axis indicates that biofilm robustness is not merely a function of matrix quantity but of structural quality, which varies independently of total biomass.

Overall, the PCA highlights a fundamental biological trade-off meaning that under optimal conditions (25 °C), the community balances planktonic growth with matrix investment, whereas under thermal stress (42 °C), the physiological profile shifts toward a degradation-mediated matrix signature with high eDNA release and low viability; these relationships are confirmed quantitatively by the pairwise correlation structure of the scaled data.

### 3.3. Structural Components of LS Community Biofilms

**Figure 7:**
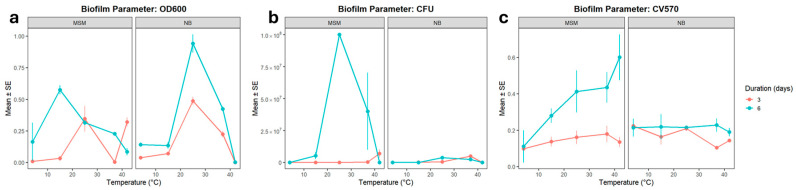
(**a**) Planktonic growth rate (OD600), (**b**) planktonic cell viability (CFU) and (**c**) attachment of the LS biofilms (expressed as crystal violet retention, measured at 570 nm) in function of temperature for LS-type MMC biofilms in function of temperature, medium, and duration of biofilm development. Bars indicate SE. n = 3 for all treatments.

LS communities showed a clear thermal optimum for planktonic growth at 25 °C, while surface attachment peaked under extreme thermal stress (Figure 7). Planktonic growth, reflected in OD600 and CFU counts, peaked significantly at 25 °C (*p* < 0.001). However, at 42 °C a distinct divergence emerged. While optical density remained high, nutrient-rich medium significantly suppressed growth (*p* < 0.001), suggesting high metabolic turnover but low population stability. Attachment (CV570) was strongly enhanced by prolonged incubation (*p* < 0.001) and reached its maximum at 42 °C in minimal medium. This suggests that under submerged (LS) conditions, higher temperatures act as a powerful trigger for surface colonization, likely as a protective response to thermal stress.

**Figure 8:**
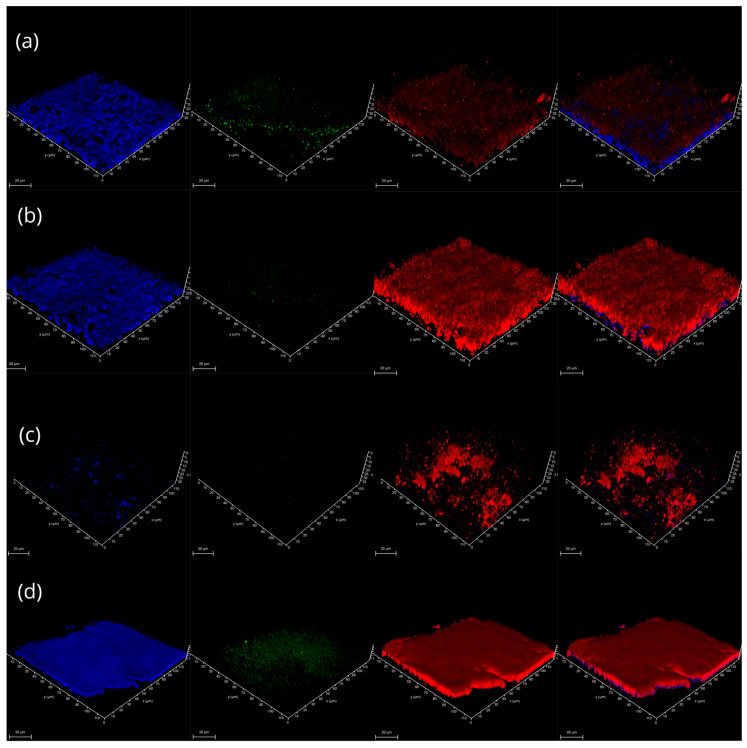
Visualized structure of LS biofilms, after 6 days of cultivation in NB medium: (**a**) 15 °C; (**b**) 25 °C; (**c**) 37 °C; (**d**) 42 °C. The red signal corresponds to total DNA, green to eDNA, and blue to cellulose, visualized with Calcofluor White. The plot at the far-right side represents the merged signal. Scale bar is 20 μm.

**Figure 9:**
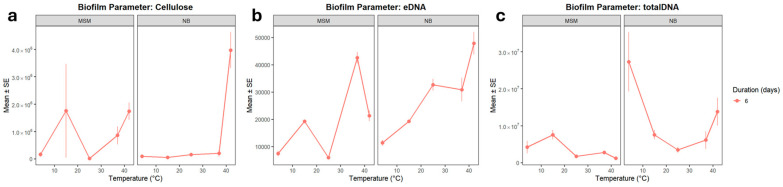
Composition of (**a**) cellulose, (**b**) eDNA, and (**c**) total DNA of the LS biofilms in function of temperature and growth medium, after six days of biofilm development. Quantification in terms of pixels in the CLSM image analysis. Bars indicate SE. n = 3 for all treatments.

Upon examination of the composition of the LS biofilms, only 6-day-old biofilms (Figure 8 and Figure 9) could be analysed due to their fragile structure after just three days of incubation. The biochemical architecture of 6-day-old LS biofilms was dominated by a marked increase in DNA and cellulose at elevated temperatures. Cellulose production was significantly elevated at 42 °C across both media types (*p* < 0.001), providing a rigid structural base for the submerged biofilm under heat stress. Both eDNA and total DNA showed a strong positive response to temperatures of 37 °C and 42 °C. The high correlation between cellulose and eDNA (R = 0.473) in LS biofilms suggests that these components co-precipitate to form a stable submerged matrix, a pattern that differs from the more independent regulation observed in ALS biofilms.

The PCA (73.8% of variance explained) revealed a fundamental trade-off between planktonic expansion and matrix investment (PC1). Thus, the planktonic/attachment axis revealed moderate warming in MSM shifted the community toward high turbidity and cell viability.

The Matrix/DNA Axis showed that high-temperature conditions in NB medium pushed the samples toward a matrix-rich signature. The decoupling of optical density from culturable biomass (PC3) further highlights that in LS communities, high turbidity at elevated temperatures does not always translate to viable or attached cells, reflecting a state of high environmental stress. Pairwise comparison of the different parameters in a pairwise plot (Figure S2) shows that cellulose content is positively correlated with eDNA (R = 0.473), but not total DNA (R = 0.141). Attachment and total DNA are slightly, inversely correlated (R = −0.378).

**Figure 10:**
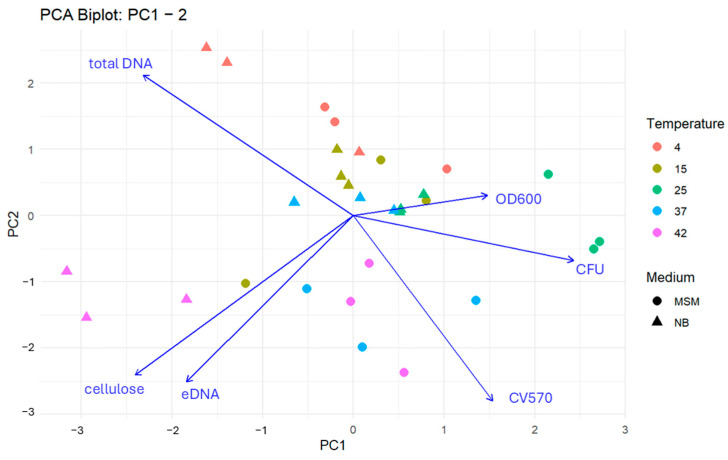
Integrated PCA-biplot for the first and second principal component illustrating the relationship of the different characteristics of LS microbial communities. Each point represents a sample characterized by cultivation temperature (colour) and medium type (shape). Arrows indicate the loadings of individual biofilm traits, with direction and length reflecting their contribution to the principal components. Traits oriented in similar directions are positively correlated, while opposing vectors suggest negative associations. Samples located near a given vector are associated with higher values of that trait.

**Figure 11:**
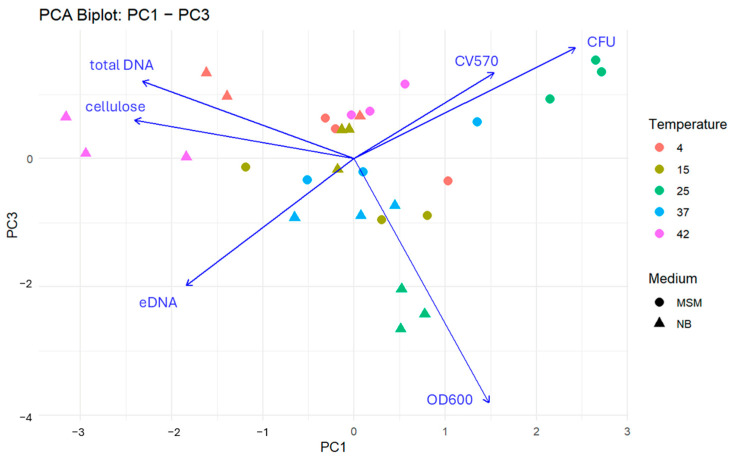
Integrated PCA-biplot for the first and third principal component illustrating the relationship of the different characteristics of LS microbial communities. Each point represents a sample characterized by cultivation temperature (colour) and medium type (shape). Arrows indicate the loadings of individual biofilm traits, with direction and length reflecting their contribution to the principal components. Traits oriented in similar directions are positively correlated, while opposing vectors suggest negative associations. Samples located near a given vector are associated with higher values of that trait.

Principal component analysis (Figure 10 and Figure 11) revealed that the six biofilm metrics can be summarized by three dominant axes, which together explain 73.8% of the total variance (PC1 = 30.6%, PC2 = 23.9%, PC3 = 19.3%). PC1 represents a clear trade-off between planktonic growth and attachment versus matrix and DNA production: OD600, CFU, and CV570 loaded positively, while cellulose, total DNA, and eDNA loaded negatively. Moderate warming in minimal media favoured a planktonic dominance, while elevated temperatures in nutrient-rich media shifted the community toward a matrix-rich signature. This indicates that under high-nutrient thermal stress, the community prioritizes the secretion of protective extracellular components over free-living growth.

PC2 (23.9%) described biomass quality though distinguishing the nature of the DNA pool within the biofilm. It contrasts total DNA content with markers of functional stability, such as eDNA and surface attachment. Notably, at 37 °C, several samples exhibited high total DNA but relatively low eDNA and attachment scores. This reveals that in submerged (LS) conditions, high cellular density does not always translate into structural integrity; rather, it may represent a crowded but weakly adhered cellular aggregation that lacks the “molecular glue” provided by eDNA.

PC3 (19.3%) highlights a decoupling between optical density (OD600) and actual cell viability (CFU and CV570). In LS communities under high-temperature stress, increased turbidity was frequently observed without a corresponding increase in culturable cells. This component serves as a biological indicator of environmental stress, where the accumulation of cellular debris and non-viable cells contributes to optical density, even as the functional biofilm population declines.

In summary, the multivariate structure of LS communities underscores a context-dependent adaptation. While 25 °C represents a physiological optimum for growth, the shift to 37–42 °C triggers a transition from a productive growth phase to a stress-driven matrix accumulation phase, characterized by structural fragility and a divergence between total biomass and community viability.

## 4. Discussion

### 4.1. Thermal Plasticity of Antarctic Plant-Associated Biofilms

Global warming and climate change are recognized as significant threats to Antarctic ecosystems, driving substantial alterations in the diversity and functionality of these specialized plant-microbial communities. These environmental changes can lead to shifts in microbial community structure and function, potentially compromising the crucial symbiotic relationships that enable Antarctic vascular plants to thrive in these environments [5]. However, the wide temperature tolerance of endophytic bacteria and the presence of the intermediate host enable those bacteria to flourish in 37–42 °C, suggesting that they possess a remarkable thermal adaptability, crucial for their survival and symbiotic functions within the fluctuating Antarctic region.

Our results demonstrate that Antarctic plant-associated biofilms possess a remarkable thermal range, extending far beyond typical maritime Antarctic summer conditions. The observed peaks in biofilm strength and attachment at 37–42 °C provide strong evidence for an ornithogenic factor in microbial dispersal. In the Antarctic Peninsula, vascular plants like *D. antarctica* and *C. quitensis* often colonize nutrient-rich soils enriched by seabird guano. The ability of these endophytes to form robust biofilms at 42 °C suggests a specialized life strategy involving birds as intermediate hosts or vectors. Interestingly, we observed a thermal switch in matrix composition. Thus, cellulose production was significantly repressed at 37 °C and 42 °C compared to the 25 °C optimum. This aligns with the known temperature-dependent regulation of the *bcs* operon in many bacteria, where cellulose production is downregulated during the transition to an endothermic host. This suggests that while these bacteria require a rigid cellulose framework for desiccation resistance on rock and plant surfaces (4–25 °C), they shift toward a more flexible, DNA-rich matrix architecture when residing within a warm-blooded host. This high-temperature “stress state” is further evidenced by the PCA (PC3), which revealed a decoupling of optical density from culturable biomass, indicating that at thermal extremes, matrix accumulation may serve as a protective response for a declining viable population.

Overall, these findings demonstrate that biofilm formation in Antarctic isolates is not governed by a single factor but emerges from the interplay of temperature, medium, and incubation duration, with each trait reflecting distinct ecological strategies for persistence and colonization outside the native cold niche.

### 4.2. Comparison Between ALS and LS Biofilms

The distinct responses of ALS and LS communities reflect the micro-environmental heterogeneity of the plant endosphere. ALS biofilms, which mimic aerated spaces like stomatal cavities, exhibited high structural stability and a synchronized response to nutrients and temperature. Their reliance on a balanced matrix of cellulose and eDNA likely protects the bacteria from fluctuating moisture levels on aerial plant surfaces.

In our studies representing submerged environments, LS communities showed a more fragile initial structure and a decoupled relationship between biomass (total DNA) and viability (CFU). The high correlation between cellulose and eDNA at 42 °C in LS biofilms (R = 0.473) suggests that submerged communities prioritize a “co-precipitated” matrix to withstand the high metabolic turnover and potential washout in liquid-rich tissues. This divergence suggests that the same microbial pool may adopt different architectural strategies depending on whether cells colonize the gas-filled apoplast (ALS) or the nutrient-rich vascular fluids (LS).

Overall, ALS and LS communities share the capacity to expand under warming but differ in their ecological strategies. ALS emphasizes moderate warming for planktonic growth and attachment, with colony viability anchored at cold baseline conditions. LS, by contrast, shows broader but more conditional responses: growth can extend to 42 °C, colony formation is optimized at 25 °C and 37 °C, and attachment is strongly promoted by incubation but suppressed by nutrient broth at high temperature. These differences highlight community-specific pathways of biofilm regulation, reflecting distinct adaptations to environmental stress.

### 4.3. Overall Ecological Significance

The dominant Antarctic flora significantly influences soil microbial community composition and activity by secreting diverse exudates that modify edaphic properties such as pH and nutrient availability, thereby impacting microbial interactions and competition for resources [25], playing a pivotal role in shaping microbial ecology of extreme ecosystems. Our results demonstrate temperature-, nutrient-, and time-dependent response in biofilm formation. This nuanced response underscores the intricate interplay of environmental factors that influence microbial community dynamics and their functional adaptations, particularly in relation to plant–microbe interactions [26].

The plant-associated microbial communities in these regions are crucial for enhancing stress tolerance, improving nutrient and water uptake, and increasing resistance to UV-B radiation, drought, and saline conditions [6]. This suggests a co-evolutionary relationship in which plants actively select and engage with specific bacteria, thus shaping the microbial community and enhancing their resilience in harsh environments [25]. Antarctic plant-specific dominant flora possesses specific life strategies such as rapid growth, metabolic versatility, and phenotypic plasticity, enabling them to endure various environmental stresses [27]. The selective pressures of the Antarctic environment also drive the potential functional redundancy of microbial communities, ensuring the persistence of core ecological functions despite microhabitat heterogeneity [28]. This potential functional redundancy also could contribute to ecosystem stability and resilience, as multiple microbial taxa can perform similar metabolic processes vital for nutrient cycling and plant health in extreme conditions. Temperature shifts in these environments may significantly alter the composition and function of these communities, potentially impacting the ecosystem’s overall stability and the plants’ ability to thrive.

Biofilm formation within the plant endosphere and rhizosphere is considered an important adaptive strategy for microbial survival and plant interaction in Antarctic conditions, facilitating nutrient exchange and protection against environmental stressors [29]. The “conditional recovery” of biofilm attachment observed at high temperatures and rich nutrients may reflect a stress-response strategy rather than a stable ecological state [30]. In nutrient-poor Antarctic soils, microbes likely to prioritize rapid expansion, but the capacity to transiently stabilize biofilm structures under episodic thermal and nutrient perturbations could contribute to short-term community resilience [31].

The pronounced contribution of extracellular DNA (eDNA) to biofilm stability at 42 °C, independent of total biomass, suggests a potential role of eDNA as a structural component that may enhance biofilm integrity under acute stress conditions [32,33,34]. However, the ecological relevance of this mechanism in natural Antarctic endosphere and rhizosphere systems remains to be experimentally validated. In the context of ongoing climate-driven environmental change, the observed functional redundancy and thermal plasticity of Antarctic endophytes may represent traits that allow flexibility across fluctuating conditions [35,36,37,38], although their direct impact on host plant performance cannot be inferred from microcosm experiments alone. Accordingly, we frame these observations as hypotheses that warrant targeted investigation rather than as demonstrated drivers of Antarctic phytomicrobiome stability.

## 5. Conclusions

This study demonstrates that Antarctic endophytic communities exhibit significant thermal plasticity, with biofilm formation and matrix investment peaking at non-native temperatures of 37–42 °C. These findings suggest that these microbes are not merely psychrotolerant survivors but are evolutionarily adapted to utilize endothermic organisms, such as seabirds, as intermediate hosts or dispersal vectors. The observed “thermal switch” characterized by the repression of cellulose and the compensatory release of eDNA at higher temperatures highlights a sophisticated reallocation of metabolic resources during the transition between environmental and host niches. However, while our synthetic community models provide valuable mechanistic insights, they are limited by the absence of natural host-tissue complexity and stochastic variations in cell viability. Future research should focus on in planta studies and the role of horizontal gene transfer within these biofilms to further elucidate the functional resilience of the Antarctic phytomicrobiome in a rapidly warming climate.

## Figures and Tables

**Table 1:** Composition of MMCs used in the study, with the numbers referring to the strains as described in [10].

MMC Type	Bacterial Strain	Host Plant
ALS	*Siminovitchia terrae* 9.1	*D. antarctica*
	*Pseudomonas salomonii* 10.1	*C. quitensis*
	*Pseudomonas yamanorum* 24.4	*D. antarctica*
	*Hafnia psychrotolerans* 25.2	*D. antarctica*
	*Pseudomonas* sp. 26.2	*D. antarctica*
	*Pseudarthrobacter* sp. 26.7	*D. antarctica*
LS	*Psychrobacter arcticus* 10.4	*C. quitensis*
	*Arthrobacter psychrochitiniphilus* 15.6	*D. antarctica*
	*Agreia* sp. 23.2	*D. antarctica*
	*Brachybacterium* sp. 39.12	*C. quitensis*
	*Kocuria salsicia* 40.1	*D. antarctica*

## Data Availability

Data will be made available at Zenodo.

## Supplementary Materials

The following supporting information can be downloaded at: https://www.mdpi.com/article/10.3390/microorganisms14030580/s1, Figure S1: Pairwise comparison plot for the ALS-type MMC biofilms. The matrix displays pairwise relationships among measured biofilm parameters across all samples. Diagonal panels show the distribution of each trait, while lower triangle panels present scatterplots illustrating bivariate associations. Upper triangle panels report Pearson correlation coefficients, indicating the strength and direction of linear relationships. For every comparison, n =−30. *: *p* < 0.05; ***: *p* < 0.001; Figure S2: Pairwise comparison plot for the LS-type MMC biofilms. The matrix displays pairwise relationships among measured biofilm parameters across all samples. Diagonal panels show the distribution of each trait, while lower triangle panels present scatterplots illustrating bivariate associations. Upper triangle panels report Pearson correlation coefficients, indicating the strength and direction of linear relationships. For every comparison n = −30. *: *p* < 0.05; ***: *p* < 0.001.

## Abbreviations

The following abbreviations are used in this manuscript:

ALS	air–liquid–solid
CFU	colony-forming units
CLSM	confocal laser scanning microscope
CV570	biofilm attachment measure
eDNA	extracellular DNA
LMM	linear mixed model
LS	liquid–solid
MSM	minimal salt medium
MMC	model microbial community
NB	nutrient broth
PC	principal component
PCA	principal component analysis
REML	restricted maximum likelihood
