# Supplementary material for: Temperature-Dependent Biofilm Development in Antarctic Endophytic Microbial Communities
Source: Microorganisms. 2026 Mar 4;14(3):580. doi: 10.3390/microorganisms14030580 (PMC13029002; doi:10.3390/microorganisms14030580)
Supplement: Supplementary file 1 [file microorganisms-14-00580-s001.zip › microorganisms-4046788-supplementary.pdf]

Supplementary Materials:

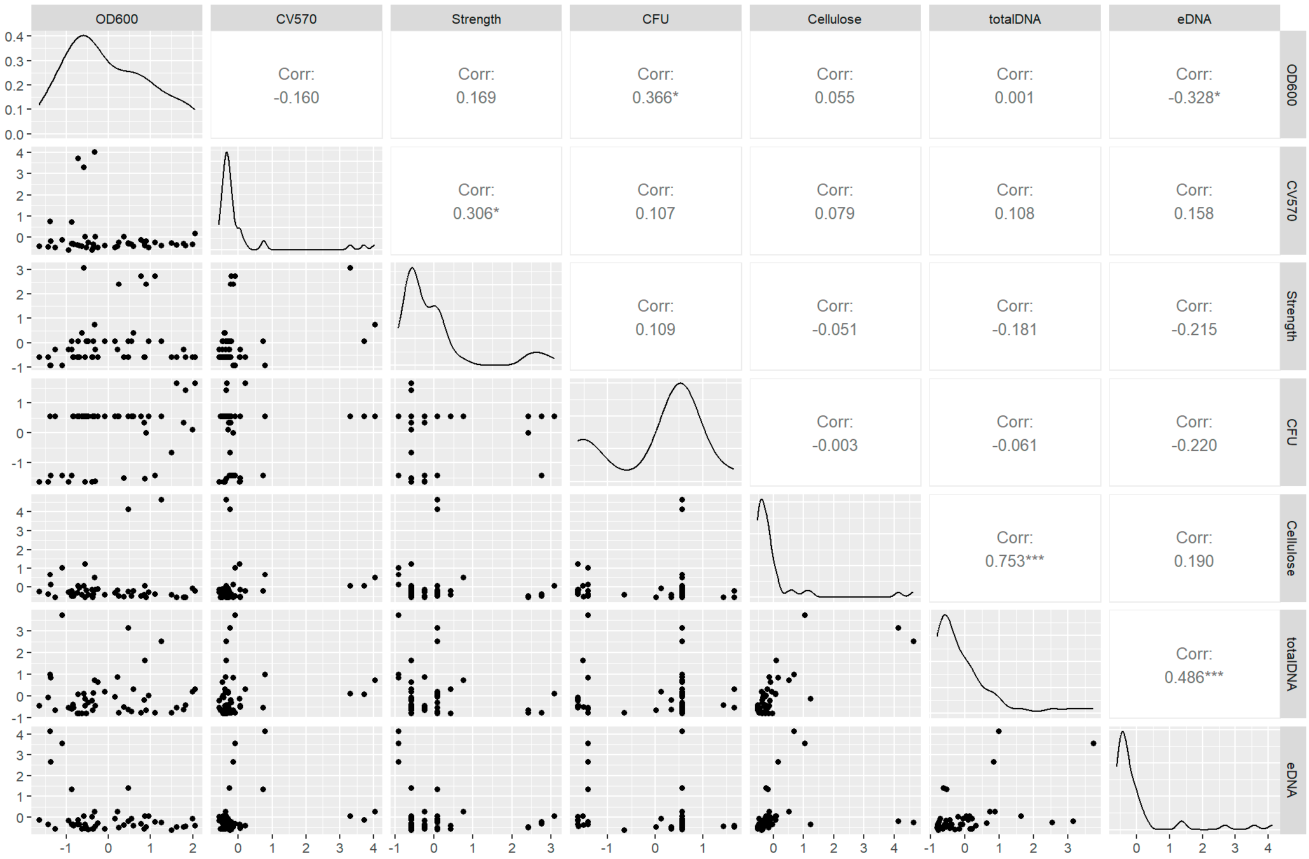

Figure S1. Pairwise comparison plot for the ALS-type MMC biofilms. The matrix displays pairwise relationships among measured biofilm parameters across all samples. Diagonal panels show the distribution of each trait, while lower triangle panels present scatterplots illustrating bivariate associations. Upper triangle panels report Pearson correlation coefficients, indicating the strength and direction of linear relationships. For every comparison, n =-30. *: $p < 0.05$; ***: $p < 0.001$.

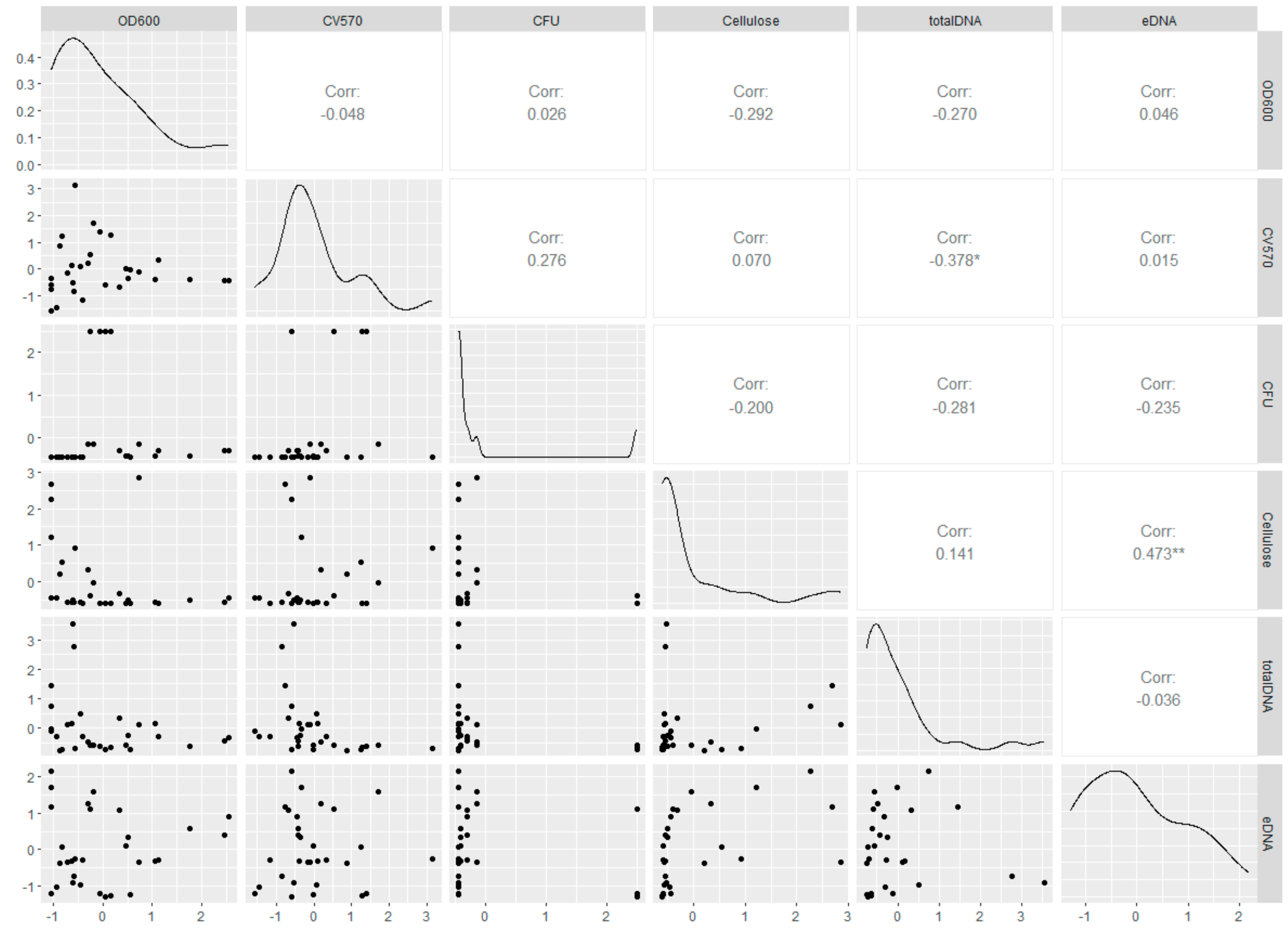

Figure S2. Pairwise comparison plot for the LS-type MMC biofilms. The matrix displays pairwise relationships among measured biofilm parameters across all samples. Diagonal panels show the distribution of each trait, while lower triangle panels present scatterplots illustrating bivariate associations. Upper triangle panels report Pearson correlation coefficients, indicating the strength and direction of linear relationships. For every comparison, n =-30. *: $p < 0.05$; ***: $p < 0.001$.
